# The detrimental impacts of negative age stereotypes on the episodic memory of older adults: does social participation moderate the effects?

**DOI:** 10.1186/s12877-020-01833-z

**Published:** 2020-11-05

**Authors:** Stephen C. Y. Chan, Alma M. L. Au, Simon M. K. Lai

**Affiliations:** 1 The Open University of Hong Kong, Good Shepherd Street, Ho Man Tin, Kowloon, Hong Kong; 2 The Public and Social Policy Research Centre of The Open University of Hong Kong, Kowloon, Hong Kong; 3 The Hong Kong Polytechnic University Hung Hom, Kowloon, Hong Kong

**Keywords:** Age stereotypes, Episodic memory, Older adults, Social participation

## Abstract

**Background:**

Older adults’ cognitive abilities can be impaired through priming of negative age stereotypes. However, it is unclear whether the effects of negative priming can be extended to episodic memory, which is believed to be the most age-sensitive type among the long-term memory systems, in Asian populations. Social participation has recently emerged as a potential protective factor for maintaining the cognitive function of older adults. The purpose of this study was to examine the effects of negative age stereotype priming on episodic memory and the moderating role of social participation in the priming effect.

**Methods:**

A total of 105 community-dwelling older adults residing in Hong Kong were randomly allocated to two experimental conditions. Participants were primed either with negative age stereotype words (*n* = 53) or neutral words (*n* = 52) using an implicit priming task. Episodic memory performance was assessed using the Hong Kong List Learning Task (HKLLT), which includes total learning, two delayed recalls and a recognition task. Analysis of covariance (ANCOVA) was used to assess group differences in the priming task and memory performance, while a series of moderation analyses were performed to examine the moderating effects of social participation.

**Results:**

The group that received negative age stereotype priming performed significantly worse than the group that received neutral words in their episodic memory test. Additional analyses showed that socially active individuals might be less prone to the effects of negative age stereotypes for the recognition task only.

**Conclusions:**

Older adults who are more socially active might be more immune to the effects of negative age stereotype priming on episodic memory. These results provide initial support for the hypothesis that social participation might act as an effective strategy to ward against negative age stereotype priming.

**Trial registration:**

ClinicalTrials.gov: NCT04202120 (first posted December 17, 2019), (Retrospectively registered).

**Supplementary Information:**

The online version contains supplementary material available at 10.1186/s12877-020-01833-z.

## Background

Age stereotypes are beliefs concerning features of the aged population [1]. They could be refined and amplified across the life span and could be manifested in both positive (e.g., wise and generative) and negative forms (e.g., unproductive and forgetful) [2]. These cognitive representations are powerful in impacting aged individuals once they feel and perceive themselves as the stereotyped group.

It is crucial to distinguish concepts between stereotype threats and priming with age stereotypes as they have been shown to affect differential outcomes in experimental designs. Jamieson and Harkins [3] alluded that stereotype threats induce motivation to disconfirm the negative stereotypes, while stereotype priming leads to a worse performance because participants withdraw their efforts in outcome measures.

The role of age stereotypes in memory performance among older individuals has been investigated with different methods. Priming has become one of the prevalent ways to activate such age stereotypes. The activation of certain stereotypes could be initiated either explicitly or implicitly in experiments. For example, negative age stereotypes were implicitly activated through a scrambled sentence task [4] or a priming task that was framed as a reaction time task [5]. The effects of implicit activation of age stereotypes tended to be more powerful [5]. Experimental studies generally suggested that the activation of negative age stereotypes has detrimental effects on performances across various domains such as physiology [6]; physical ability in terms of walking ability [7]; and more importantly, memory performances [5].

The impacts induced by implicit activation of negative age stereotypes could be explained by Stereotype Embodiment Theory (SET). With reference to SET, age stereotypes are internalized by assimilating them with the corresponding culture across the life span, and they would become salient from self-relevance [8]. The internalization of age stereotypes has been shown to be associated with differential outcomes such as physical and functional health [9, 10], subjective well-being [11], physical activity and health-seeking behavior [12] and the will to live and survive [13].

Horton and colleagues [14] conducted a meta-analysis investigating the effect of priming age stereotypes on aged adults and the overall weighted effect size yielded *d* = .38. Among different conditions, it was found that the negative age stereotype exerts an effect nearly three times greater than the positive age stereotype does, despite both types impacting performance measures [15]. Furthermore, the implicit priming effect was found to be more powerful than the explicit priming effect on memory performance and more salient when stereotyping cues are task-relevant [16, 17]. Based on the review, although both positive and negative age stereotypes could be activated, the effects of negative age stereotypes on memory performances outweigh those of positive age stereotypes. Thus, we only included negative age stereotype primes and neutral word primes for the priming task in the current study.

To replicate the findings of previous studies, we chose to investigate episodic memory by using The Hong Kong List Learning (HKLLT) as it is a comprehensive tool allowing researchers to dissect performance differences at different stages [18].

Cultural differences in the prevalence of age stereotypes have also been found in the literature. Generally, it is reported that the perception of older adults tends to be more positive in the East than the West due to its cultural values, including Confucianism and respect [19]. Although Asia culture emulates Confucius in humbleness, compliance and harmony, some studies have shown that older people living in urban settings rated themselves more negatively in self-perception of aging than did others [20]. The emerging negative view could be attributed to modernization, allowing the older adults to compare themselves with others whom they perceived as better-off. A persistent negative view of aging could further loop and strengthen a vicious cycle, which directly or indirectly promotes negative age stereotypes. Thus, it could be asserted that negative age stereotypes are rampant in Asian culture.

Increasing amounts of evidence from studies has supported the hypothesis that cognitive performances could be influenced by psychosocial factors [21]. It is argued that social systems provide opportunities for psychosocial mechanisms, including social support and social engagement that impact individuals’ behaviorally, psychologically and physiologically [22]. Highly socially integrated individuals are able to develop positive self-perceptions of aging. It has been found that older adults with a more negative perception of aging would tend to participate less in society [23]. In contrast, socially active individuals could develop a larger social network and make more social contacts with others. Those who possess a variety of interests in various activities also reported more positive self-perceptions of aging [24]. Social engagement or social participation is believed to be one way for older adults to facilitate socialization and develop positive emotional and cognitive well-being. Thus, older adults with higher social participation rates are expected to possess fewer negative age stereotypes, and its concomitant effects would be reduced. Concurrently, it is predicted that the priming manipulation should have less of an impact on cognitive performances among relatively socially active participants.

As reviewed, previous studies have been done on consolidating the relationship between priming negative age stereotypes and performance outcomes, yet there are far fewer studies in the Asian context where societal values about older individuals are changing [25]. In this study, we examined the effects of negative aging stereotype priming on episodic memory using an implicit priming task. To the best of our knowledge, this is the first experiment using an implicit priming task to examine the effects of aging stereotypes on memory performance among older adults in Hong Kong. It also suggests a potential indicator, social participation, as a buffer against these negative age stereotypes.

With reference to the above literature, it is thought that negative age stereotypes are present in older adults in Hong Kong. We first predicted that aged adults primed with negative aging stereotypes would perform worse on the memory task than the control group. Specifically, we predicted that aged adults in the experimental group would learn less well, recall fewer words on the delayed recall session as well as recognize fewer words in the recognition task than their control counterparts. Moreover, it is postulated that the relationship between priming manipulation and memory performance would be moderated by social participation as operationalized by the frequency of joining activities.

## Methods

### Participants

Most of the participants were members of the Institute of Active Ageing of the Hong Kong Polytechnic University. The experiment was first promoted by the institute using its internal email system and participants were recruited through phone calls. Other participants were recruited by referrals through snowball sampling. This study targeted individuals aged 60 years or above. The recruited participants were generally well-educated, physically healthy (self-reported), mentally healthy (using The Montreal Cognitive Assessment (MoCA)) and were able to read or speak fluent Chinese/Cantonese. A prior power analysis was performed and determined that a total sample size of 92 subjects was required to have 95% power for detecting an effect size of .38 (as reviewed in meta-analysis) when taking α = .05. Thus, 110 participants were drawn from the recruiting pool to take part in the study to avoid data loss.

Participants were blinded to the assignment of conditions and were randomly assigned, according to their number of participation in the trial using simple randomization, to either the neutral word priming group (control group) or the negative age stereotype priming group (experimental group). The research team generated random numbers for the random assignment procedure. Participants were given a $200 (~USD$25) supermarket coupon after completion of the experiment.

### Design

The present study was a between-subject design where participants were randomly assigned to one of two conditions.

The priming task is adopted and modified from a previous study [5, 26]. It was performed using E-prime 2.0 software [27]. To ensure the primes flashed on screen were beyond awareness, a similar adjustment procedure was used (see [26]). The individualized stimulus onset asynchrony (SOA) was determined in each trial block in which a total of ten neutral words was flashed either 1 cm above or 1 cm below the cross-point (center) in each trial. The participants were required to focus on the cross-point and to respond to the computer by pressing the designated keys as quickly and accurately as possible. Patterned masks (rows of at signs: @) were used before and after each flash of a word. After the trial block, participants were asked to try to report any words viewed during each trial. Their SOA were reduced or enhanced whenever 2 words or above were correctly reported or no single word could be reported, respectively. The priming SOA for this study ranged from 32 ms to 208 ms (M = 98.70 ms, *SD* = 48.60).

Negative age stereotype primes were taken from Levy’s study while neutral words were adopted from the most frequent words used in the Chinese context [5]. To ensure the words are relevant to the Hong Kong elderly population, a list of 60 words (22 words are positive, 22 words are negative and 16 words are neutral) was generated, and then 16 participants between 50 and 69 years old rated each of the words according to its relevance to themselves in a 7-point Likert type scale ranging from 1 indicating *‘very positively related to you’* to 7 *‘very negatively related to you’* while the score of 4 referred to *‘irrelevant to you’*. The negative words and neutral words rated at the highest frequency were used in the priming task.

This study extended the priming procedure in which 4 blocks of 40 trials were presented to the individualized SOA. In the negative age stereotype priming condition, 40 trials consisted of (i) 12 negative age stereotype primes (repeated once); (ii) 2 highly rated negative age stereotype primes (i.e., dementia and clumsy) (repeated twice); (iii) 4 selected neutral words (besides, sentence, moreover and even) (repeated once). More specifically, 12 negative age stereotype primes and 4 neutral words were presented twice and 2 highly rated negative age stereotype primes were presented four times, which constitutes 24 trials and 8 trials, respectively, out of 40 trials in each block. In the neutral condition, 4 blocks of the 40 neutral words were flashed randomly.

The priming procedure was intensified based on Levy’s (1996) priming paradigm as we would such as to exacerbate the priming effects as well as counterbalance the number of trials in each of the blocks to avoid fatigue and the tendency of pressing the same key.

**Fig. 1:**
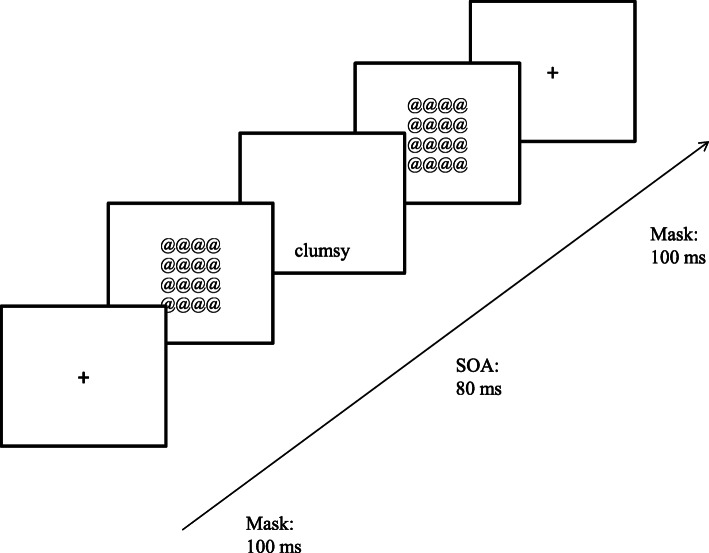
An example of the flow of the masked priming for one typical trial

During each trial, similar to the prior individualized procedure, the participant was asked to indicate whether the stimulus was flashed above or below the cross-point. The typical flow of each trial is shown in Fig. 1.

After responding to the 40th trial in each block, four emotional words (two negative and two positive) were randomly presented to the participants and they were asked to rate whether the targeted words were positive or negative. As suggested by previous studies, individuals who were negatively primed would tend to respond to the negative words in a quicker manner, which could be seen as the activation of a negative stereotype at the subliminal level [28]. The reaction time and correct rate of clicking the flashes were presented to the participant after finishing the whole task.

### Measures

Montreal Cognitive Assessment (MoCA) was used as a baseline assessment. It is a 10-min test that evaluates several cognitive domains with a total score of 30. The Hong Kong version is validated and is available at the MoCA official website [29].

The HKLLT is a validated tool for assessing episodic memory for aged Chinese-speaking adults [18]. It is also used for investigating differences among older adults with normal cognitive ability and older adults with mild cognitive impairment [30]. A random control list was used in this study. It is comprised of 16 words formed by four categories: *family member (grandmother)*, *country (Chile)*, *furniture (wardrobe)* and *vegetables (cucumber)*. All words were in random order such that no words within the same category were presented consecutively. Three attempts were presented to the participants and the total learning score (out of 48) was computed over three trials. It also involved 10- and 30-min delayed recalls as well as a recognition task. The recognition task required participants to indicate in list of 32 items, where half of them were targets while half of them were foils, in a yes or no manner. A discrimination score was calculated as it considers both correct hits and false alarm errors.

Sociodemographic information and a scale measuring social participation were included in a questionnaire. Self-rated health was measured using a single item ranging from 1 (‘*very good’*) to 5 (‘*very poor’*). Expenditure was captured by the perceptual item ‘Do you have enough money for daily expenditures?’ Participants were asked to rate this item ranging from 1 ‘*very insufficient’* to 5 ‘*more than enough’*. Social participation was measured by a list of 10 activities based on the proposed inventory (see Additional file 1) [31]. Participants were asked to indicate the frequency of each activity on a 6-point Likert-type scale ranging from 0 (‘*never’*) to 5 (‘*always’*) within the past month. The average score of this scale was used for analysis, with a higher score indicating a higher rate of social participation.

### Procedure

**Fig. 2:**
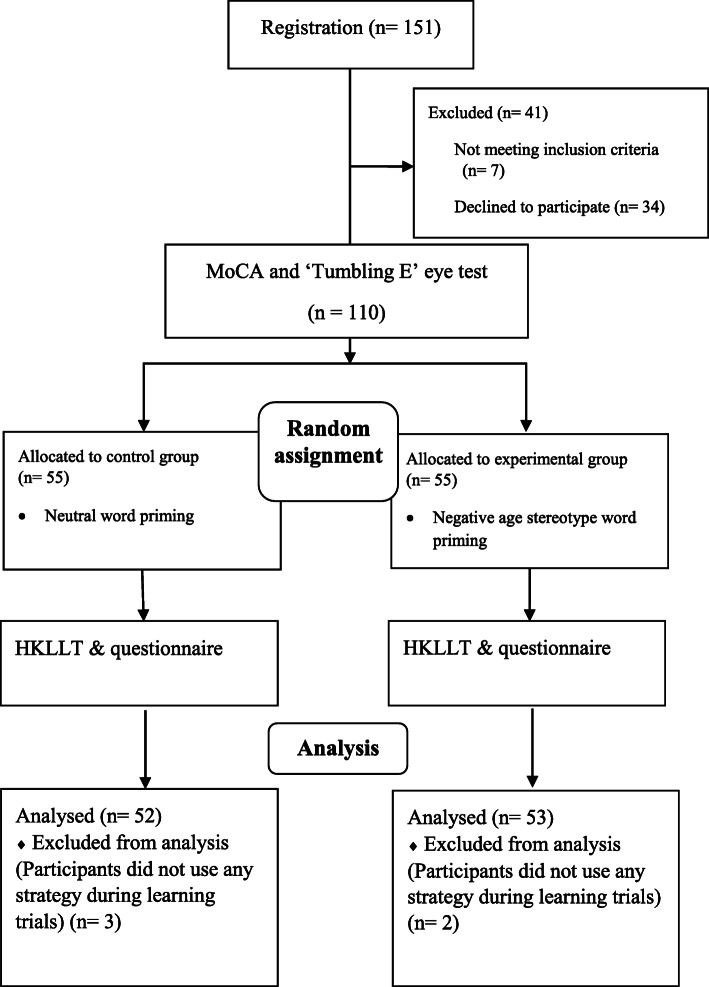
Research flow of the present study

The experiment was done in the laboratory setting and it was divided into three sections: (i) prepriming test, (ii) the priming task & (iii) memory assessment. The experiment lasted for approximately 2 h and its research flow is presented in Fig. 2. Participants first signed the written informed consent, and then took the MoCA and a simple visual acuity test using the “Tumbling E” Eye Chart. The simple visual acuity test was chosen to ensure participants did not possess severe problems with their eye-sight so that they were able to perform the priming task on the computer screen [32]. Then, participants entered the trial session for determining their personal SOA before the priming stage. This stage was framed as a reaction time test in which they were asked to press the appropriate key as accurately and as quickly as possible using their individualized SOA. This deceptive explanation was also put on the information sheet in order to avoid contamination of the present priming task. Participants started the priming task according to the allocated randomized treatment.

The HKLLT was implemented immediately after the priming task. All task instructions were standardized and computerized according to the manual of HKLLT by using E-prime 2.0. During the first learning trial, participants first listened to the 16 target words and were asked to record the words aloud to the microphone. No feedback was given by the examiner or the computer. The procedure was repeated in the second and third learning trials. Participants were also asked whether they had been using any strategic methods to memorize the words.

After the learning trials in the HKLLT, participants were asked to fill out a questionnaire including sociodemographic information and other scales such as frequency of social participation. Without prior notification, the participants were asked to stop filling out the questionnaire and asked to recall the list of words again after 10 min and then 20 min (i.e., 30-min delayed recall). The recognition task was then immediately performed. The remaining time was given for completion of the questionnaire if necessary. After signing the receipt of coupon collection, the debriefing session was given in which the research flow was explained. Participants were also asked not to reveal any details of the study.

### Planned analyses

Analyses were performed using SPSS, version 25. Multiple independent samples *t* tests and chi-square independence tests were used to investigate differences in sociodemographic characteristics and baseline assessments. Analysis of covariance (ANCOVA) was used to test group differences in priming manipulation and memory performance.

Since the 10-min and 30-min delayed recall scores were highly correlated (*r* = .89), we computed one composite score (total delayed recall) by adding the two delayed recall scores. The total learning score, total delayed recall and discrimination score in the recognition trial were taken as dependent variables while the average score of social participation frequency was taken as a moderator for the moderation analyses using the SPSS macro PROCESS (model 1) [33]. This is taken for analyses since it allows researchers to use continuous variables for moderators as well as for probing the interaction, if any, by using the Johnson-Neyman (JN) technique.

## Results

### Baseline measures

#### Sociodemographic variables

We recruited 151 potential participants into this study, but 41 of them were excluded as they did not meet the inclusion criteria (aged below 60) or declined to participate. The remaining pool of participants was evenly and randomly assigned to either the experimental condition or control condition. Five participants in both groups were excluded from the analysis as they reported that they did not use any strategy during learning trials in the memory test.

No significant differences between the groups were found. The sociodemographic information of the primed group is presented in Table 1. Age, sex, education years and health status were taken as covariates for further analyses.

**Table 1:** Sociodemographic information of the participants across the groups

	Experimental group (*n* = 53)		Control Group (*n* = 52)		Statistics			
	Percentage	Mean (*SD*)	Percentage	Mean (*SD*)	*t*	*χ2*	*df*	*p*
Age		65.40 (3.10) (range: 61–71)		65.15 (2.55) (range: 56–71)	−.44	/	103	.66
Sex (Male)	50.9%		48.1%		/	.09	1	.77
Education (in years)		12.96 (2.67)		13.46 (2.85)	.93	/	103	.36
Marriage (Married)	69.8%		78.8%		/	1.12	1	.29
Income ($)					/	6.47	3	.09
< 6000	47.2%		34.6%					
6000–14,999	34%		26.9%					
15,000–24,999	7.5%		25.0%					
25,000 or above	11.3%		13.5%					
Job status					/	1.23	1	.27
Retired	83%		90.4%					
Unemployed or with a part-time job	17%		9.6%					
Expenditure (out of 5)		3.19 (.86)		3.42 (.70)	1.54	/	103	.13
Self-rated health (out of 5)		3.11 (.78)		2.87 (.93)	−1.48	/	103	.14
Social participation (out of 5)		2.48 (.70)		2.65 (.64)	1.31	/	103	.19
MoCA score		27.30 (1.34)		27.81 (1.55)	1.79	/	103	.08

#### Montreal cognitive assessment (MoCA)

The MoCA is used as a brief and potential screening tool for detecting Mild Cognitive Impairment (MCI) and Alzheimer’s disease (AD) and it has been validated in Hong Kong. A score of less than 22 is considered as a cut-off in Hong Kong [34]. All participants passed this cut-off (*M* = 27.55, *SD* = 1.46). There is a component assessing the delayed recall in the MoCA test. The results indicated that there was no significant difference between the priming group and the control group for this component [*t* (92.57) =1.48, *p* = .142]. However, there was a marginally significant difference between the two groups on the overall MoCA score [*t* (103) =1.79, *p* = .076], and hence, the overall MoCA score was controlled as a covariate for further analyses.

#### Priming manipulation

As the priming task was framed as a reaction task, the percentage of correct hits and response time are shown at the end of the task. The individualized SOA might act as a critical factor in affecting the priming task and its reaction time for hitting the targets. Referring to Table 2, there were no differences found in the manipulation of the personalized SOA [*t* (103) = − 1.30, *p* = .198], the percentage of correct hits of the target during priming [*t* (84.01) = .191, *p* = .061] as well as the reaction time for hitting each priming target [*t* (103) = −.30, *p* = .766].

**Table 2:** Priming information across groups

	Experimental group (*n* = 53)	Control Group (*n* = 52)	Statistics			
	Mean (*SD*)	Mean (*SD*)	*t*	*F*	*df*	*p*
Individualized SOA (ms)	103.24 (41.34)	92.92 (40.21)	−1.30	/	103	.198
Correct hits (%) ^a^	97.66 (2.78)	98.50 (1.62)	1.91	/	84.01	.060
Reaction time (ms)	497.03 (129.91)	490.10 (106.22)	−.30	/	103	.766
Reaction time to negative emotional words	974.45 (154.94)	1053.03 (212.52)	/	5.81	97	.018
Reaction time to positive emotional words	996.35 (258.31)	816.12 (137.76)	/	18.52	97	<.001

Note

^a^Degrees of freedom were adjusted due to violation of the homogeneity assumption

#### Emotional word reaction time

As discussed, it is reasoned that the time responding to negative words will be shortened if negative primes are activated in the experimental group. Our finding of an average reaction time to emotional words supported this argument after controlling for the baseline reaction time, MoCA score and sociodemographic variables. The ANCOVA results suggested that the experimental group reacted significantly faster to negative emotional words than did the control [*F* (1, 97) = 5.81, *p* = .018, ƞp^2^ = .06], indicating a possibility of activating negative age-stereotypes during the priming task. More interestingly, they also reacted significantly slower to positive emotional words compared to the control group [*F* (1, 97) = 18.52, *p* < .001, ƞp^2^ = .16].

**Fig. 3:**
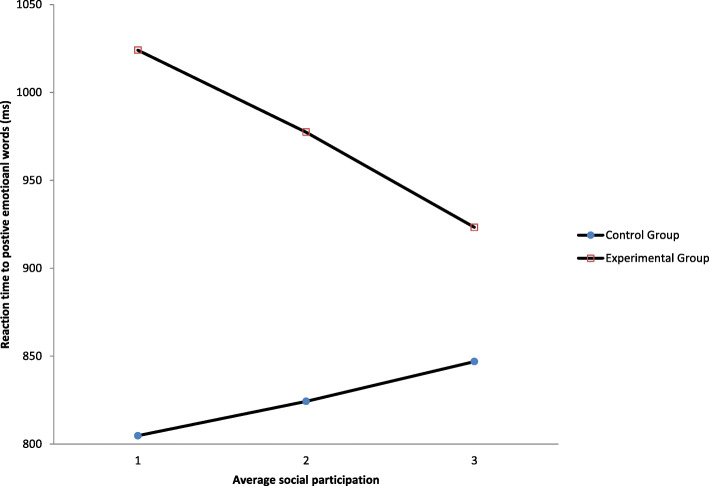
Moderation effect of social participation between experimental group and reaction time to positive emotional words

Further analyses were conducted to investigate whether social participation would moderate the effects of the priming task by controlling for the baseline reaction time, MoCA score and other covariates of the individuals. Referring to Table 3, there was a significant main effect of priming manipulation on reaction time to positive emotional words after controlling for all covariates (*B* = 438.14, *t* = 3.14, *p* = .003, CI =153.27, 723.00). As shown in Fig. 3, the interaction effect of priming manipulation and reaction time to positive emotional words by social participation was found to be significant (*B* = − 109.62, *t* = 2.04, *p* = .045, CI = − 216.57, − 2.66), suggesting that participants in the experimental condition who were relatively less socially active showed a longer reaction time in rating positive emotional words. All the regression weights were found to be nonsignificant in the relationship between the experimental condition and reaction time to negative emotional words as well as in the moderation analysis.

**Table 3:** Moderation analyses of social participation between experimental conditions and reaction time to emotional words

Outcome: Reaction time to positive emotional words, *R*^2^ = .24, MSE = 41,759.88						
Variables	*B*	*SE*	*t*	*p*	LLCI	ULCI
Experimental group	438.14	143.49	3.05	.003**	153.27	723.00
Social participation	32.34	40.48	.799	.426	−48.03	112.71
Experimental group*Social participation	−109.62	53.88	−2.04	.045*	−216.57	−2.66

*Note*. *p < .05, **p < .01, ***p < .001

#### Memory performance in HKLLT

Scoring and clinical findings of the HKLLT could be retrieved from previous studies [35]. The mean scores of each learning trial, total learning scores, 10-min and 30-min delayed recall as well as the discrimination score of the recognition task are shown in Table 4. Controlling the baseline MoCA score and other covariates, the control group learned significantly more words in the total learning trials [*F* (1, 98) = 15.01, *p* < .001, ƞp^2^ = .13], recalled more words in the 10- and 30-min delayed recall [*F* (1, 98) = 46.86, *p* < .001, ƞp^2^ = .33; *F* (1, 98) = 48.14, *p* < .001, ƞp^2^ = .33] and performed better in the recognition task than the experimental group [*F* (1, 98) = 14.55, *p* < .001, ƞp^2^ = .13]. Furthermore, the rate of forgetting in the first 10 min was computed by the suggested formula as [(10-min delayed recall – learning trial 3)/learning trial 3 × 100%] [35]. ANCOVA results indicated that the experimental group made more intrusion errors [*F* (1, 98) = 28.64, *p* < .001, ƞp^2^ = .23] and had a significantly higher rate of forgetting [*F* (1, 98) = 14.66, *p* < .001, ƞp^2^ = .13] than the control group.

**Table 4:** Memory performance across groups

	Experimental group (*n* = 53)	Control Group (*n* = 52)	Statistics			
	Mean (*SD*)	Mean (*SD*)	*F*	*df*	*p*	ƞ_p_^2^
Learning trial 1	6.36 (1.77)	7.23 (1.91)	3.44	98	.07	.03
Learning trial 2	9.43 (1.81)	11.04 (2.14)	14.47	98	<.001	.13
Learning trial 3	11.23 (2.02)	12.90 (1.67)	17.02	98	<.001	.15
Total learning	27.02 (4.72)	31.17 (5.06)	15.01	98	<.001	.13
10-min delayed recall^a^	8.81 (2.33)	11.90 (2.14)	46.86	98	<.001	.32
Rate of forgetting	−20.95 (19.83)	−7.97 (9.77)	14.66	98	<.001	.13
30-min delayed recall^a^	8.79 (2.59)	12.08 (2.31)	48.14	98	<.001	.33
Total intrusion errors	4.17 (2.99)	1.38 (1.57)	28.64	98	<.001	.23
Discrimination score of recognition	77.12% (17.07)	89.18% (9.68)	14.55	98	<.001	.13

*Note*. ^a^out of 16 words

#### Moderation analyses of memory performances

**Fig. 4:**
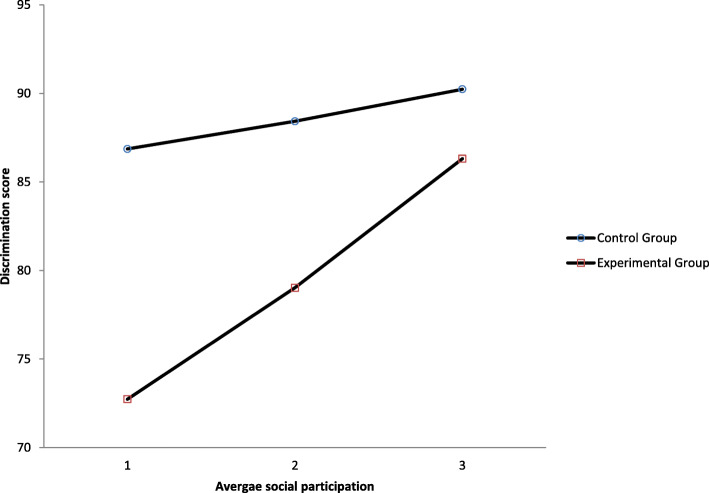
Moderation effect of social participation between experimental group and recognition trial performance

Table 5 shows the results of the moderation analyses between the experimental condition and the memory performances. The interaction effects of priming manipulation on total learning and total recall by social participation were nonsignificant (*p*s > .05). However, there was an interaction effect of priming manipulation by social participation found in the discrimination score in the recognition trial (*B* = 7.83, *t* = 2.13, *p* = .036, CI = .52, 15.14), suggesting that participants who were relatively less socially active performed worse compared to their counterparts. In other words, those who were negatively primed but socially active did relatively equal to the control group as shown in Fig. 4. Taking the Johnson-Neyman (JN) technique to probe this interaction, the output identified individuals with a .71 standard deviation on social participation (24.76% of our sample was above this) would not be affected by the negative priming manipulation.

**Table 5:** Moderation analyses of social participation between experimental conditions and memory performances

Outcome: Discrimination score, *R*^2^ = .39, MSE = 150.87						
Variables	*B*	*SE*	*t*	*p*	LLCI	ULCI
Experimental group	−29.75	9.81	−3.03	.003**	−49.22	−10.29
Standardized social participation	2.59	2.77	.94	.352	−2.91	8.08
Experimental group*Social participation	7.83	3.68	2.13*	.036*	.52	15.14

*p < .05, **p < .01, ***p < .001

## Discussion

This study examined how implicitly priming negative age stereotypes could induce detrimental effects on memory performance among older adults in Hong Kong, and our results replicated those of previous studies, as reviewed. Participants were primed with negative aged-based stereotypes in the experimental group and these stereotypes were assumed to be activated at a subliminal level as indicated by the shorter reaction time to negative emotional words but a longer reaction time to positive emotional words in the experimental group.

Concerning the priming manipulation, socially less active participants who were primed with negative age stereotypes showed a longer reaction time in rating positive emotional words. However, for negative emotional words, there was an absence of a moderating effect. It is possible that after activation of their own negative perceptions of aging, when they were asked to rate positive emotional words, those who were relatively active were assumed to possess less negative age stereotypes so that the response time to positive emotional word remained similar, but those who were less active and with a more negative view of aging might need to spend cognitive resources on counteracting or reducing self-doubt and worries [36]. Moreover, it could also be argued that when negative age stereotypes were activated, presenting contradictory words (i.e., positive emotional words) might saliently induce self-doubt, thus reduced the availability of resources for performing reaction time tasks as well as the subsequent memory tasks.

Our study also supported our hypothesis that negative age stereotypes could impact the memory performances of older adults. The present results were consistent with studies conducted in both Asian and Western countries [5, 37]. HKLLT is an essential tool for dissecting the whole process of a memory task, including the stages of learning, recalling and recognizing. Throughout the procedures, our results consistently demonstrated that the experimental group did worse than the control group in all stages after controlling for their baseline performances and other covariates. It could be argued that negative age-based stereotype primes would interfere with working memory in all stages. The availability of resources for performing the memory tasks was reduced, and therefore, they learned worse than their counterparts, even when sematic organization was used as an effective strategy. Furthermore, it could be assumed that they performed worse as they withdrew efforts after the activation of age stereotype priming [3]. This underlying mechanism might be reflected by the triad results among the experimental group of a higher forgetting rate, fewer correctly recalled words as well as a greater chance of making intrusion errors.

Our results partially supported the hypothesis that participants who were negatively primed but with greater social participation would be less affected, although this effect was significant only in the relationship between priming manipulation and recognition scores. It could be possible that negative age stereotypes are too compelling after the priming task, so that during the learning and recall sessions, participants might spend cognitive resources to resist or cope with the withdrawal effect. However, after a period of time (i.e., an hour after the priming task), the experimental effect faded and those who were socially active might shake off the activation effects of negative age stereotypes, and they performed almost as well as the control subjects.

Another possible interpretation is related to the nature of the memory test. The present studies showed that the magnitude of the effect was stronger in the delayed-recall tasks than the recognition tasks. Recall and recognition involve differential mechanisms in processing and the recognition task is generally considered to be less demanding [38]. The delayed recall process is a more self-initiated process that demands more cognitive efforts relative to the recognition tests, which rely more on situational and environmental cues. Research generally supports that both processes involve brain activation in the area of the right prefrontal cortex and the anterior cingulate, yet, compared to recall, recognition has higher activation in the right inferior parietal cortex, providing evidence that both processes are involved in the two modes of episodic retrieval [38]. The activation of negative age stereotypes might exert a greater negative force on the recall task; this might explain why the moderation effects were nonsignificant in the delayed recall tasks but became significant in a recognition task that was relatively less demanding [39].

A large body of research has indicated the positive impacts of social participation on the physical and psychological well-being of aged adults. Engaging in productive and social activities are found to be the key variables associated with subjective and psychological well-being among older individuals [40]. The number of social activities participated in was also found to be significantly and positively associated with mental status and memory [41]. Furthermore, the quality of the social activity participation also played an essential role in enhancing the quality of life of the elderly after retirement [42, 43]. Another study reported that the elderly who participated more frequently in community social activities scored lower on the Negative Ageing Stereotypes Assessment Questionnaire, and the researchers argued that the elderly who participate less in daily life are more prone to suffering from negative beliefs about social contacts, which further promotes the risk of suffering from health, cognitive and mental problems [24]. This study might provide a possible intervention, namely, that social participation might act as an effective strategy against negative age stereotypes.

Our study contributes to the literature as the number of studies using subliminal experimental conditions of age stereotypes is meager. Comparable and more systematic results are presented in our study by analyzing the whole learning, recalling and recognition processes. Moreover, we also-modified and extended the priming procedures as we believed that the degree or duration of stereotype threat exposure might affect the memory performances to a certain extent. Our results showed that the experimental group did worse on both delayed recall tests and recognition tests, and this might serve as a preliminary evidence for a longer duration of exposure to negative age stereotypes inducing relatively stronger effects, leading to higher detrimental impacts on both tests. Future studies could be carried out on how negative age stereotypes could affect memory performances by varying the intensity and/or duration or primes.

There are a few limitations of the current study. One of them is that our participants are relatively well educated and they all scored relatively highly on the MoCA test. A previous study indicated negative age stereotypes could be more destructive among younger elderly people and among participants with a higher education, although it was using stereotype threat manipulation [4], so the strong effect of priming manipulation in this study could be justified. However, those who are older and with a relatively lower education background might need further investigation.

Another limitation concerns our findings. Although there was a preliminary result supporting the hypothesis that relatively socially active aged adults were less likely to be impacted in the memory test, this effect was found to be significant only in the recognition task of the memory test. There is the possibility that socially active individuals could possess fewer negative age stereotypes, but the effects would be more or less similar once some saliently particular negative age stereotypes were activated. Future studies could try to explore whether there could be specific negative age stereotype primes that exert specific impacts on performance outcomes.

## Conclusions

To sum up, by including a neutral group, this study replicated and extended the investigation of how negative age stereotype priming affects the whole process of memory performance using implicit priming manipulation among aged adults in Hong Kong. We also added a potential indicator, frequency of social participation, as a moderator of this effect. That is, social participation might not just act as a booster for the well-being of aged adults, but it also might be a potential strategy to ward against the detrimental effects of negative age stereotype priming.

## Supplementary Information

**Additional file 1.** Ten items for measuring social participation.

## Data Availability

The datasets used and/or analyzed during the current study are available from the corresponding author on reasonable request.

## Abbreviations

SET: Stereotype Embodiment Theory
MoCA: Montreal Cognitive Assessment
SOA: Stimulus onset asynchrony
HKLLT: The Hong Kong List Learning Test
ANCOVA: Analysis of covariance
AD: Alzheimer’s disease
