# Supplementary material for: The detrimental impacts of negative age stereotypes on the episodic memory of older adults: does social participation moderate the effects?
Source: BMC Geriatr. 2020 Nov 5;20:452. doi: 10.1186/s12877-020-01833-z (PMC7643323; doi:10.1186/s12877-020-01833-z)
Supplement: Supplementary file 1 — Additional file 1. Ten items for measuring social participation. [file 12877_2020_1833_MOESM1_ESM.docx]

**Appendix A.** Ten items for measuring social participation

| Levels | Activity domain | Items | Ratings |
| --- | --- | --- | --- |
| 3 | Informal social activity | 1) Physical meetings with friends or relatives  2) Contacting others through technological products or apps | In terms of frequency:  How often do you participate in the following activities?  0: Never  1: Occasionally (about once/month)  2: Quite often (about once/ bi-weekly)  3: Often (about once/week)  4: Usually (about 2-4times/ week)  5: Quite always (more than 4 times/week) |
| 4 | Leisure activity | 3) Physical activities  (E.g. Sports)  4) Cultural activities  (E.g. Watching Chinese opera)  5) Recreational activities  (E.g. joining a party or dinner with friends)  6) Religious activities (E.g. attending church) |  |
| 5 | Productive activity | 7)Caregiving  8)Voluntary work |  |
| 6 | Formal social activity | 9) Social organizational activities  10) Political or civic activities (E.g. political forum) |  |
